# [^18^F]fluorodeoxyglucose positron emission tomography/computed tomography characteristics of primary mediastinal germ cell tumors

**DOI:** 10.1038/s41598-023-44913-x

**Published:** 2023-10-17

**Authors:** Koeun Lee, Yong-il Kim, Jungsu S. Oh, Seung Yeon Seo, Jae Kwang Yun, Geun Dong Lee, Sehoon Choi, Hyeong Ryul Kim, Yong-Hee Kim, Dong Kwan Kim, Seung-Il Park, Jin-Sook Ryu

**Affiliations:** 1 Department of Nuclear Medicine, Kangbuk Samsung Hospital, Sungkyunkwan University School of Medicine, Seoul, Republic of Korea; 2 Department of Nuclear Medicine, Asan Medical Center, University of Ulsan College of Medicine, Seoul, Republic of Korea; 3 Department of Thoracic and Cardiovascular Surgery, Asan Medical Center, University of Ulsan College of Medicine, Seoul, Republic of Korea

**Keywords:** Oncology, Cancer imaging

## Abstract

Primary mediastinal germ cell tumor (MGCT) is an uncommon tumor. Although it has histology similar to that of gonadal germ cell tumor (GCT), the prognosis for MGCT is generally worse than that for gonadal GCT. We performed visual assessment and quantitative analysis of [^18^F]fluorodeoxyglucose positron emission tomography/computed tomography ([^18^F]FDG PET/CT) for MGCTs. A total of 35 MGCT patients (age = 33.1 ± 16.8 years, F:M = 16:19) who underwent preoperative PET/CT were retrospectively reviewed. The pathologic diagnosis of MGCTs identified 24 mature teratomas, 4 seminomas, 5 yolk sac tumors, and 2 mixed germ cell tumors. Visual assessment was performed by categorizing the uptake intensity, distribution, and contour of primary MGCTs. Quantitative parameters including the maximum standardized uptake value (SUVmax), tumor-to-background ratio (TBR), metabolic tumor volume (MTV), total lesion glycolysis (TLG), and maximum diameter were compared between benign and malignant MGCTs. On visual assessment, the uptake intensity was the only significant parameter for differentiating between benign and malignant MGCTs (*p* = 0.040). In quantitative analysis, the SUVmax (*p* < 0.001), TBR (*p* < 0.001), MTV (*p* = 0.033), and TLG (*p* < 0.001) showed significantly higher values for malignant MGCTs compared with benign MGCTs. In receiver operating characteristic (ROC) curve analysis of these quantitative parameters, the SUVmax had the highest area under the curve (AUC) (AUC = 0.947, *p* < 0.001). Furthermore, the SUVmax could differentiate between seminomas and nonseminomatous germ cell tumors (*p* = 0.042) and reflect serum alpha fetoprotein (AFP) levels (*p* = 0.012). The visual uptake intensity and SUVmax on [^18^F]FDG PET/CT showed discriminative ability for benign and malignant MGCTs. Moreover, the SUVmax may associate with AFP levels.

## Introduction

Primary mediastinal germ cell tumors (MGCTs) are uncommon tumors that account for 2–4% of all germ cell tumors (GCTs), which may be found predominantly in young males with markedly elevated tumor markers such as alpha fetoprotein (AFP) or human chorionic gonadotropin (HCG)^1,2^. Due to histological resemblance, primary MGCTs can be classified in the same way as GCTs. Mature teratoma is generally considered as benign MGCT, and malignant MGCTs can be broadly subdivided into seminomas and nonseminomatous germ cell tumors (NSGCTs)^3,4^. Different NSGCT entities include immature teratoma, seminoma, yolk sac tumor, choriocarcinoma, and embryonal carcinoma. When more than one type of histology is present, the tumor may be considered as mixed MGCT (malignant MGCT entity). Although MGCTs have similar histology similar to that of gonadal GCTs, the prognosis for MGCTs is generally worse than that for gonadal GCTs; however, studies of MGCTs have shown that patients with seminomas have a more favorable prognosis than that of patients with NSGCTs^5^.

There have been no prospective studies to define the diagnostic approach, prognostic stratification, and treatment strategies for MGCTs, and only a few retrospective studies and case series exist^6^. Therefore, the treatment and management of this disease remain challenging and require a multidisciplinary approach. The tumor location, extension, serum tumor markers, and histopathological type are considered critical information for the diagnosis of and treatment planning for GCTs^7^.

Recently, the role of [^18^F]fluorodeoxyglucose positron emission tomography/computed tomography ([^18^F]FDG PET/CT) in the diagnosis and prognostic stratification of mediastinal tumors has been investigated^8,9^. However, there are limited [^18^F]FDG PET/CT-related studies on primary MGCTs, and those that have been published mainly consist of case reports and small series. A few studies reported that [^18^F]FDG PET/CT had additional value for detecting distant metastasis or recurrence after chemotherapy^10,11^. Other studies reported that [^18^F]FDG PET/CT parameters showed positive associations with tumor markers such as AFP and HCG^12,13^. In another preliminary study, the tumor-to-mediastinal ratio of [^18^F]FDG PET/CT was found to be significantly correlated with the expression of Glut1, HIF-1, EGFR, p-Akt, and p-S6K in primary non-thymic neoplasms^14^. However, no structured study has conducted about the visual assessment or quantitative analysis of [^18^F]FDG PET/CT parameters for MCGTs.

We examined the usefulness of visual assessment and quantitative analysis of [^18^F]FDG PET/CT in primary MGCT differentiation. In addition, we investigated the association between serum tumor markers and quantitative PET/CT parameters.

## Results

### Patient characteristics

A total of 35 consecutive patients (16 females and 19 males) were included in the analysis. The median patient age at the time of PET/CT image acquisition was 27 years. The median time interval between biopsy or surgery and PET/CT was 13 days (range, 0–134 days). The patients were finally pathologically diagnosed with MGCTs through excisional biopsy (n = 24) or resective surgery (n = 11). A total of 24 patients had benign mature teratomas, and 11 patients had malignant lesions, including seminomas (n = 4), yolk cell tumors (n = 5), and mixed GCTs (n = 2) (Table 1).

**Table 1:** Clinical and pathological characteristics.

Variables	Total
Number	35
Age (years)	33.1 ± 16.8 (15–77, median 27)
Sex (F:M)	16:19
Time interval between surgery and PET/CT (days)	29.5 ± 36.6 (0–134, median 13)
Alpha fetoprotein (AFP, ng/mL)	2044.4 ± 6290.8 (0.6–30,800.0, median 1.85) [26*]
Human chorionic gonadotrophin (HCG, mIU/mL)	12.5 ± 42.5 (1.0–197.0, median 1.0) [26*]
Clear resection margin	32 (91.4%)
Lymph node metastasis	2 (5.7%)
Pathologic diagnosis	
Mature teratoma	24 (68.6%)
Seminoma	4 (11.4%)
Yolk sac tumor	5 (14.3%)
Mixed germ cell tumor	2 (4.7%)

Continuous parameters were expressed as mean ± standard deviation with range for normal distribution and included median when the values were non-normal distribution.

*Number of patients with tumor marker data that were included in the analysis.

In comparison with patients with benign MGCTs, patients with malignant MGCTs were significantly younger (38.2 ± 18.0 years vs. 21.9 ± 4.0 years, *p* < 0.001). All 11 patients in the malignant group were male. The time interval between surgery and PET/CT was not significantly different between benign and malignant MGCTs (*p* = 0.221). The tumor markers AFP and HCG were examined in 26 of the 35 patients, and AFP was found to be significantly higher in patients with malignant MGCTs than in patients with benign MGCTs (*p* = 0.004), whereas HCG showed no significant difference (*p* = 0.604) (Table 2).

**Table 2:** Comparison of clinical and pathological characteristics between benign and malignant MGCTs.

Variables	Benign MGCT (n = 24)	Malignant MGCT (n = 11)	*p-*value
Age (years)	38.2 ± 18.0 (15–77)	21.9 ± 4.0 (18–31)	< 0.001*
Sex (F:M)	16:8	0:11	< 0.001*
Time interval between surgery and PET/CT (days)	28.5 ± 34.3 (0–134, median 16)	31.6 ± 42.9 (0–111, median 10)	0.221
AFP (ng/mL)	7.2 ± 22.2 (0.6–87.3, median 1.0) [15^†^]	4822.5 ± 9177.2 (1.1–30,800.0, median 16.8) [11^†^]	0.004*
HCG (mIU/mL)	14.1 ± 50.6 (1.0–197.0, median 1.0) [15^†^]	10.4 ± 30.4 (1.0–102.0, median 1.0) [11^†^]	0.604
Clear resection margin	24/24 (100.0%)	8/11 (72.7%)	0.025*
Lymph node metastasis	0/24 (0.0%)	2/11 (18.2%)	0.092

*MGCT* mediastinal germ cell tumor, *AFP* alpha fetoprotein, *HCG* human chorionic gonadotrophin.

Continuous parameters were expressed as mean ± standard deviation with range for normal distribution and included median when the values were non-normal distribution.

*p < 0.05.

^†^Number of patients with tumor marker data that were included in the analysis.

### Visual assessment

All 10 grade 1 or 2 cases involved benign MGCTs, and all malignant MGCTs showed grade 3 uptake. The uptake intensity could differentiate between benign and malignant MGCTs (*p* = 0.040). The distribution and contour were not significant in differentiating between benign and malignant MGCTs (*p* = 0.146 and 0.331, respectively) (Table 3).

**Table 3:** Comparison of visual assessment between benign and malignant MGCTs.

Variables	Grade or characteristics	Total (n = 35)	Benign MGCT (n = 24)	Malignant MGCT (n = 11)	*p*-value
Uptake intensity	Grade 1	6	6	0	0.040*
	Grade 2	4	4	0	
	Grade 3	25	14	11	
Distribution	Homogenous	6	6	0	0.146
	Heterogenous	29	18	11	
Contour	Round	26	20	6	0.331
	Lobulated	8	4	4	
	Infiltrative	1	0	1	

*MGCT* mediastinal germ cell tumor.

Uptake grading: grade 1, uptake equal or less than liver; grade 2, uptake greater than liver.

*p < 0.05.

### Quantitative analysis

**Figure 1:**
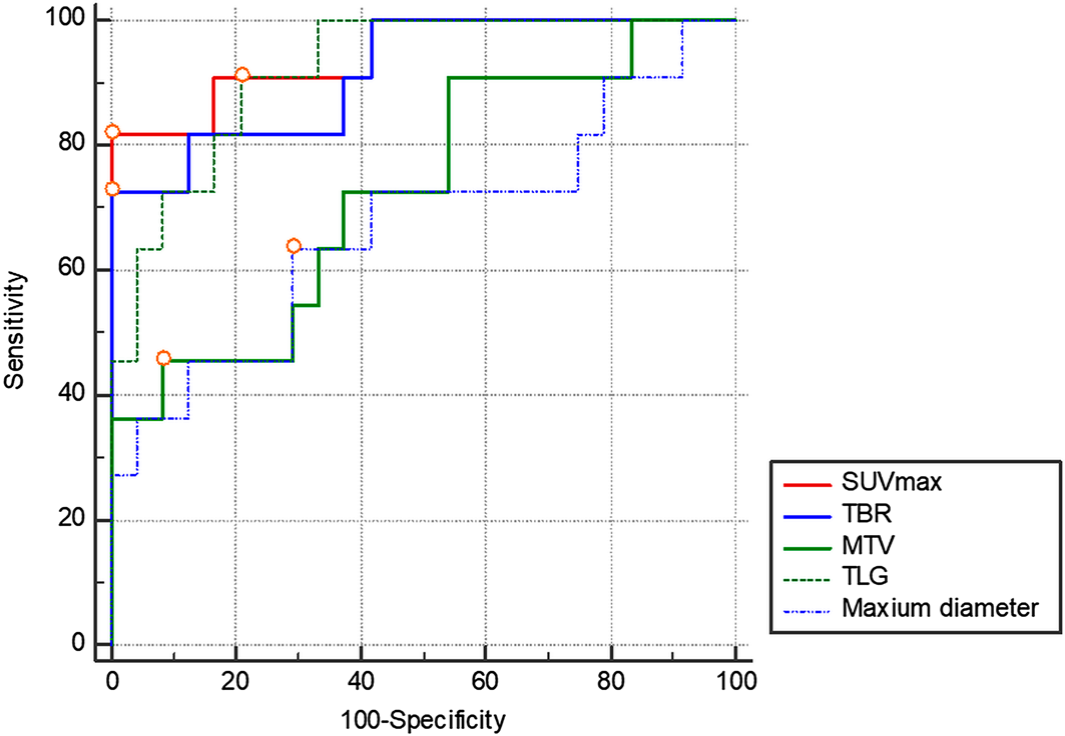
Receiver operating characteristic (ROC) curve analysis of PET/CT parameters. In ROC curve analysis, all PET parameters had a larger area under the curve (AUC) compared with that of the maximum diameter, and the SUVmax had the largest AUC of 0.947.

The median values of the PET/CT parameters of all patients were SUVmax 3.3, TBR 3.2, MTV 12.6 cm^3^, and TLG 18.8. The PET/CT parameters SUVmax, TBR, MTV, and TLG were significantly higher in the malignant MGCT group than in the benign MGCT group (*p* < 0.001,* p* < 0.001, *p* = 0.033, and *p* < 0.001, respectively), whereas the maximum diameter did not show a significant difference (*p* = 0.115) between the two groups (Table 4 and Fig. 1).

**Table 4:** Comparison of quantitative parameters between benign versus malignant MGCTs and seminomas versus malignant NSGCTs.

PET/CT parameters	Total (n = 35)	Benign MGCT (n = 24)	Malignant MGCT (n = 11)	*p-*value	Seminoma (n = 4)	NSGCT (n = 7)	*p-*value
SUVmax	4.9 ± 4.7 (0.5–18.7, median 3.3)	2.5 ± 1.2 (0.6–4.5, median 2.4)	10.3 ± 4.9 (3.2–18.7, median 10.7)	< 0.001*	6.3 ± 2.5 (3.2–9.1, median 6.5)	12.5 ± 4.6 (3.7–18.7, median 13.2)	0.042*
TBR	5.0 ± 5.2 (0.4–23.3, median 3.2)	2.5 ± 1.4 (0.4–4.9, median 2.3)	10.2 ± 6.5 (2.9–23.3, median 9.7)	< 0.001*	5.7 ± 2.7 (2.9–9.0, median 5.6)	12.8 ± 6.7 (3.0–23.3, median 10.3)	0.042*
MTV (cm^3^)	24.9 ± 42.4 (0.7–215.3, median 12.6)	12.5 ± 10.3 (0.7–50.2, medina 10.6)	51.9 ± 68.5 (4.3–215.3, median 16.2)	0.033*	11.3 ± 3.5 (7.7–14.6, median 11.5)	75.2 ± 78.0 (4.3–215.3, median 52.9)	0.073
TLG	153.6 ± 415.4 (0.3–1862.7, median 18.8)	20.7 ± 22.3 (0.3–107.1, median 15.4)	443.5 ± 671.4 (18.0–1862.7, median 84.4)	< 0.001*	48.5 ± 29.3 (18.0–84.4, median 45.9)	669.1 ± 766.5 (28.5–1862.7, median 408.1)	0.109
Maximum diameter (cm)	8.5 ± 3.5 (2.9–18.5)	7.7 ± 2.7 (2.9–12.6)	10.7 ± 4.4 (4.4–18.5)	0.036	6.4 ± 2.1 (4.4–9.4)	12.7 ± 3.7 (7.9–18.5)	0.341

*MGCT* mediastinal germ cell tumor, *NSGCT* nonseminomatous germ cell tumor, *SUVmax* maximum standardized uptake value, *TBR* tumor-to-background ratio, *MTV* metabolic tumor volume, *TLG* total lesion glycolysis.

Continuous parameters were expressed as mean ± standard deviation with range for normal distribution and included median when the values were non-normal distribution.

*p < 0.05.

**Figure 2:**
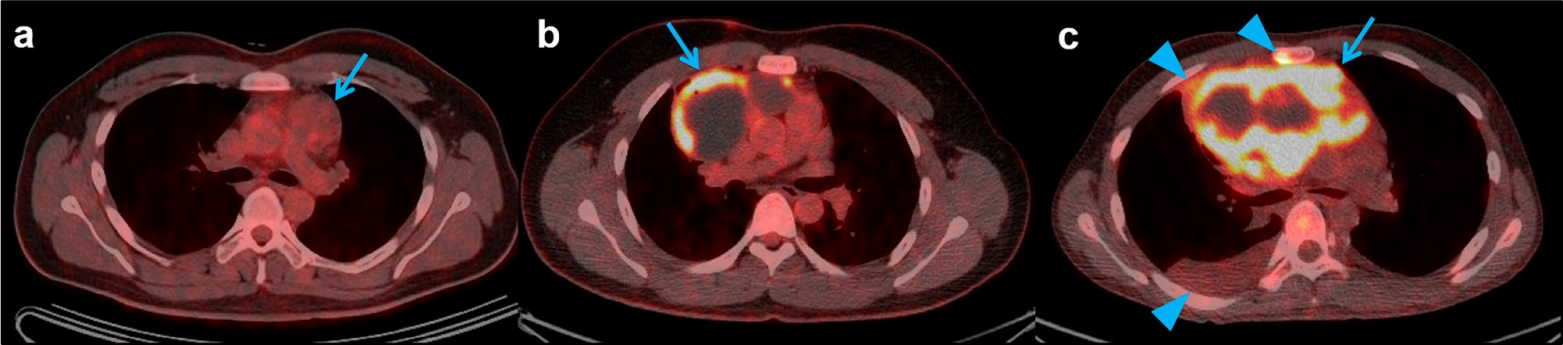
Representative ^18^F-FDG PET/CT cases of mediastinal germ cell tumors (MGCTs) with the pathologic subtype. (**a**) A 21-year-old man with benign MGCT (mature teratoma) showing grade 1 uptake, homogenous distribution, and round contour (SUVmax 1.9, arrow). (**b**) A 31-year-old man with seminoma showing grade 3 uptake, heterogenous distribution, and lobulated contour (SUVmax 15.0, arrow). (**c**) A 22-year-old man with yolk cell tumor showing grade 3 uptake, heterogenous distribution, and infiltrative contour (SUVmax 18.7, arrow) with anterior pleural invasion, pleural effusion, and bone metastasis in the sternum (arrowheads).

In ROC curve analysis of the evaluated parameters, the SUVmax had the highest AUC (AUC = 0.947, cut-off > 4.5) and demonstrated significantly better diagnostic performance than that of the MTV and maximum diameter in discriminating between benign and malignant MGCTs. A specific cut-off value of 4.5 for the SUVmax showed sensitivity of 81.8% and specificity of 100.0% in differentiating between benign and malignant MGCTs. The TBR (AUC = 0.917, cut-off > 4.9) and TLG (AUC = 0.920, cut-off > 24.6) also showed significant discriminative performance in differentiating between benign and malignant MGCTs; however, the AUC was not significantly different compared with that of the SUVmax (Table 5 and Fig. 2).

**Table 5:** ROC curve analysis of quantitative parameters in differentiating benign and malignant MGCTs.

PET/CT parameters	AUC	Cut-off value	*p-*value	Sensitivity (%)	Specificity (%)	AUC comparison with SUVmax
SUVmax	0.947	> 4.5	< 0.001*	81.8	100.0	
TBR	0.917	> 4.9	< 0.001*	72.7	100.0	0.222
MTV (cm^3^)	0.727	> 20.3	0.020*	45.5	91.7	0.025^†^
TLG	0.920	> 24.6	< 0.001*	90.9	79.2	0.484
Maximum diameter (cm)	0.670	> 8.8	0.129	63.6	70.8	0.011^†^

*ROC* receiver operating characteristics, *AUC* area under curve, *SUVmax* maximum standardized uptake value, *TBR* tumor-to-background ratio, *MTV* metabolic tumor volume, *TLG* total lesion glycolysis.

*p < 0.05.

^†^Significant difference of AUC compared with the SUVmax.

### Comparison of quantitative parameters between seminomas and NSGCTs

Malignant lesions were subdivided into seminomas and NSGCTs for subgroup analysis. Among the evaluated parameters, the SUVmax, TBR, and maximum diameter showed significant differences between the two subgroups (*p* = 0.042, *p* = 0.042, and *p* = 0.012, respectively) (Table 4).

### Association between quantitative parameters and tumor markers

Patients were divided into two groups (high and low) according to the threshold of the median value of each tumor marker (AFP = 2 and HCG = 1). The SUVmax, TBR, and maximum diameter were significantly higher in the high AFP group (*p* = 0.012, *p* = 0.034, and *p* = 0.044, respectively) (Table 6); however, no PET/CT parameters showed significant differences between the high and low HCG groups (Supplementary Table 1).

**Table 6:** Analysis of quantitative parameters according to AFP.

Parameters	AFP < 2 (n = 13)	AFP ≥ 2 (n = 13)	*p*-value
Age (years)	24.6 ± 7.1 (15–43)	30.3 ± 18.3 (16–77, median 22.0)	0.305
Sex (F:M)	7:6	3:10	0.226
SUVmax	3.4 ± 1.8 (1.3–6.8, median 3.3)	8.7 ± 5.6 (2.4–18.7, median 9.1)	0.012*
TBR	3.5 ± 1.6 (1.4–6.7, median 3.7)	8.9 ± 6.6 (2.5–23.3, median 9.0)	0.034*
MTV (cm^3^)	16.5 ± 12.1 (5.0–50.2, median 16.4)	14.6 ± 64.6 (4.3–215.3, median 14.6)	0.614
TLG	33.7 ± 27.4 (6.2–107.0, median 31.3)	374.0 ± 635.7 (10.9–1862.7, median 44.9)	0.081
Maximum diameter (cm)	8.0 ± 2.8 (4.4–12.6)	10.8 ± 3.6 (5.9–18.5)	0.120

*AFP* alpha fetoprotein, *SUVmax* maximum standardized uptake value, *TBR* tumor-to-background ratio, *MTV* metabolic tumor volume, *TLG* total lesion glycolysis.

Continuous parameters were expressed as mean ± standard deviation with range for normal distribution and included median when the values were non-normal distribution.

*p < 0.05.

## Discussion

In this study, distinctive features of MGCTs were identified using the visual uptake intensity on [^18^F]FDG PET/CT. All malignant MGCTs showed grade 3 uptake. In addition, the information provided by the quantitative [^18^F]FDG PET/CT parameters showed potential for discriminating benign MGCTs from malignant MGCTs. All quantitative PET parameters showed significantly higher values for malignant MGCTs compared with MGCTs. Among the parameters evaluated, the SUVmax had the highest AUC value and exhibited superior diagnostic performance than that of the MTV and maximum diameter. Furthermore, the SUVmax was significantly higher for NSGCTs and the high AFP subgroup.

MGCT is a disease with heterogeneous entities, accounting for approximately 14–24% of mediastinal tumors^15,16^ with high prevalence in young adults and children^17^. The role of [^18^F]FDG PET/CT in the initial diagnosis of MGCTs has not been established, which needs to be further investigated^6^. In terms of visual analysis, a review indicated that benign MGCTs could show mild-to-moderate uptake and that malignant MGCTs could demonstrate higher uptake than that of benign MGCTs, which are consistent with our study results^18^. Based on visual observations, we found that the increased uptake of [^18^F]FDG PET/CT higher than that of the liver could be a potential sign of malignant MGCTs. In contrast, the distribution and contour showed no significant difference^19^, indicating that the uptake intensity could be a better visual assessment parameter. In terms of quantitative analysis, the SUVmax had a higher AUC value than that of volumetric parameters such as the MTV or TLG in the differentiation of malignant and benign MGCTs. Therefore, the oncologic behavior of MGCTs might be largely dependent on which malignant component of the GCT histology is included rather than the whole volume of tumor cells, such as in the case of thymic epithelial tumors^20^.

Among malignant MGCTs, seminomas account for two-thirds of tumors, and NSGCTs account for the remaining one-third^21^. The prognosis of NSGCTs is worse than that of seminomas; thus, differentiation between these two entities is important^5^. As [^18^F]FDG PET/CT reflects the glycolysis pathway, high SUVmax is usually correlated with aggressiveness and proliferative activity^22^. Our results demonstrated the possibility of discrimination between seminomas and NSGCTs using the SUVmax.

A recent study reported that for patients with elevated (at least two) tumor markers after surgery, [^18^F]FDG PET/CT had a higher positive predictive value in the primary staging of testicular GCTs^12^. The authors also reported that the MTV and TLG showed significant positive correlations with HCG, whereas AFP showed a significant correlation only for patients with NSGCTs. Similarly, our study showed significance between tumor marker levels and quantitative parameters; however, a positive association was only observed with AFP levels. Differences between the studies could be explained by the heterogeneity of patients included in each study^23^. Specifically, differences in the proportions of subtypes in each study could have led to different results considering that seminomas rarely produce AFP but may have a variable amount of HCG; in contrast, high AFP levels in yolk cell tumors and high HCG levels in choriocarcinomas are common and strongly correlated with the whole tumor volume.

This study has several limitations. First, as this was a single-center retrospective study, the possibility of referral bias cannot be excluded. Second, some of the patients did not have tumor marker results and were excluded from subgroup analysis. Finally, as our sample size was small, other tumor markers including lactate dehydrogenase, which is usually elevated in malignant MGCTs, could not be evaluated. A prospective, multicenter study with a larger number of patients is needed in the future.

In conclusion, the visual uptake intensity of MGCTs on [^18^F]FDG PET/CT was useful for discriminating between benign and malignant MGCTs. Additionally, the SUVmax on [^18^F]FDG PET/CT could provide information for differentiating between benign and malignant MGCTs. Specifically, the SUVmax could differentiate seminomas and NSGCTs among malignant MGCTs and may be associated with AFP levels.

## Methods

### Study design and subjects

**Figure 3:**
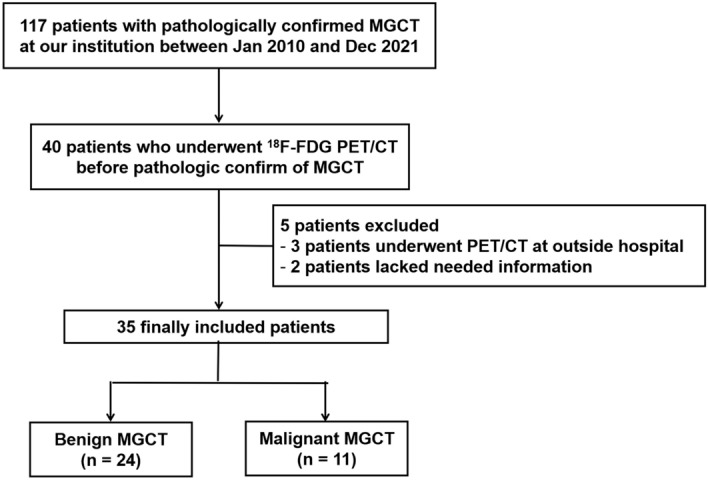
Flowchart of patient selection.

We retrospectively reviewed the medical records of 117 patients who underwent biopsy or surgery for MGCTs at our institution between January 2010 and December 2021. Among these patients, 40 patients who underwent [^18^F]FDG PET/CT within four months prior to biopsy or surgery were identified. Five patients who underwent PET/CT at an other hospital and who did not have all the information needed for the quantification of [^18^F]FDG PET/CT data (i.e., radiotracer injection dose, injection-to-scan time) were excluded from this study (Fig. 3). This study was approved by the Institutional Review Board (IRB) of Asan Medical Center (IRB no. S2021-2042-0001), and all methods were performed in accordance with the relevant guidelines and regulations. The need for informed consent was waived by the IRB of Asan Medical Center because of the retrospective nature of the study.

### [^18^F]FDG PET/CT image acquisition

All of the studied patients fasted for at least 6 h before [^18^F]FDG PET/CT image acquisition, and their venous blood glucose levels were maintained below 150 mg/dl. Patients were positioned in the scanners with their arms above their heads. [^18^F]FDG PET/CT was performed using one of the following PET/CT scanners: Biograph TruePoint 40 (Siemens Healthineers, Erlangen, Germany), Discovery 690, Discovery 690 Elite, or Discovery 710 (GE Healthcare, Milwaukee, WI, USA). The patients were intravenously administered 5.2 MBq/kg [^18^F]FDG, and PET emission images were obtained 1 h after the injection, with 5–6 bed positions covering the skull base to the upper thigh. The PET parameters included 2.5 min/bed and 168 × 168 matrix size (Biograph TruePoint 40) or 2 min/bed and 192 × 192 matrix size (Discovery series) in the 3D acquisition mode. The CT acquisition parameters were 120 kVp, 10 mA, and a 5 mm slice thickness (Biograph TruePoint 40) or 140 kVp, Auto mA, and a 3.75 mm slice thickness (Discovery series). PET images were reconstructed using a 3D ordered-subset expectation maximization (OSEM) algorithm with attenuation correction based on the CT data.

### [^18^F]FDG PET/CT image analysis

Both visual assessment and quantitative analyses were performed by two researchers (K. L. and Y-i. K.) who were blinded to the clinical information. The PET/CT images were visually assessed, and each case was categorized according to the [^18^F]FDG uptake intensity, distribution, and contour of the primary MGCT. For image interpretation, the uptake intensity grade was defined as follows: grade 1, lower or equal to mediastinal uptake; grade 2, greater than mediastinal uptake but lower or equal to liver uptake; grade 3, greater than liver uptake. The distribution was defined as homogenous (uniform uptake and shape) or heterogeneous (uneven uptake and shape). The contour was defined as round (similar width and length with smooth margin), lobulated (smooth margin), or infiltrative (irregular margin). All categories were evaluated using PET, CT, and PET/CT fusion images.

For the quantitative analysis of PET/CT images, which included data from different scanners, the SUVs were equalized across the scanners. Using an American College of Radiology-approved Esser phantom (Data Spectrum, Hillsborough, NC, USA) filled with [^18^F]FDG water solution to set the SUVs of the hot cylinders to 2.5 and the background to 1.0, the SUVs of different hot cylinders with varying diameters were measured, and recovery coefficient (i.e., SUV ratios relative to the ideal SUV of 2.5) plots were generated. Recovery coefficients allowed the estimation of the optimal smoothing kernel size for each matched recovery coefficient generated. After harmonization, the volume-of-interest (VOI) of the mediastinal mass was drawn on PET/CT fusion images, which allowed the VOI to be drawn within the mass shown on the CT images. All PET/CT parameters obtained from Biograph TruePoint 40 data were calculated by drawing one VOI using the Mirada DBx workstation (version 1.2.0.59; Mirada Medical Ltd., Oxford, UK). Harmonized SUV values from the Discovery series data were measured using the in-house software ‘AMC NM Toolkit for Image Quantification of Excellence (ANTIQUE)’ with manually traced VOIs^24–26^.

The maximum standardized uptake value (SUVmax) was the highest pixel uptake in the mediastinal mass, and the tumor-to-background ratio (TBR) was calculated as the SUVmax of the mediastinal mass divided by the mean SUV (SUVmean) of the aorta within 2 cm^3^. The volumetric parameter metabolic tumor volume (MTV) was segmented by applying a threshold of more than 50% of the relative value of the SUVmax within the VOI, and the total lesion glycolysis (TLG) was calculated as the SUVmean multiplied by the MTV. The maximum diameter of the tumor was measured on a combined transaxial CT image.

### Statistical analysis

Continuous parameters were expressed as mean ± standard deviation with range for normal distribution and included median when the values were non-normal distribution. Categorical parameters were analyzed by Pearson’s chi-square test or Fischer’s exact test to differentiate between benign and malignant MGCTs. Continuous parameters were analyzed by independent-samples t-test or Mann–Whitney *U* test. Receiver operating characteristic (ROC) curve analysis was performed to evaluate the diagnostic performance for benign and malignant MGCTs. DeLong’s method was used to compare the area under the curve (AUC) values and their 95% confidence intervals (CIs). Optimal cut-off values were defined as the exploratory cut-off values with the highest accuracy based on the Youden Index. All statistical analyses were performed using SPSS software version 17 (SPSS, Chicago, IL) and MedCalc version 19.2.3 (MedCalc Software Ltd., Ostend, Belgium).

### Supplementary Information

Supplementary Table 1.

## Data Availability

The datasets generated during and/or analysed during the current study are available from the corresponding author on reasonable request.
