# Supplementary material for: [18F]fluorodeoxyglucose positron emission tomography/computed tomography characteristics of primary mediastinal germ cell tumors
Source: Sci Rep. 2023 Oct 17;13:17619. doi: 10.1038/s41598-023-44913-x (PMC10582033; doi:10.1038/s41598-023-44913-x)
Supplement: Supplementary file 1 — Supplementary Table 1. [file 41598_2023_44913_MOESM1_ESM.docx]

**Supplementary Table 1.** Analysis of quantitative parameters according to HCG

| Parameters | HCG ≤1 (n = 18) | HCG >1  (n = 8) | *p*–value |
| --- | --- | --- | --- |
| Age (years) | 30.4 ± 15.8  (15-77, median 27) | 20.9 ± 3.4  (16-27) | 0.108 |
| Sex (F:M) | 8:10 | 2:6 | 0.420 |
| SUVmax | 5.9 ± 5.2  (1.3–18.7, median 3.6) | 6.6 ± 4.2  (1.4–13.2, median 6.5) | 0.605 |
| TBR | 6.3 ± 6.2  (1.6–23.3, median 3.8) | 5.8 ± 3.6  (1.4–10.3, median 5.6) | 0.807 |
| MTV  (cm^3^) | 37.0 ± 56.0  (4.3–215.3, median 16.5) | 17.1 ± 15.9  (4.3–52.9, median 13.2) | 0.397 |
| TLG | 257.0 ± 559.6  (10.9–1862.7, median 30.5) | 84.4 ± 132.9  (6.2–408.1, median 32.0) | 0.807 |
| Maximum  diameter (cm) | 10 ± 3.5  (4.6-18.5) | 7.9 ± 2.9  (4.4-12.6) | 0.703 |

HCG, human chorionic gonadotrophin; SUVmax, maximum standardized uptake value; TBR, tumor-to-background ratio; MTV, metabolic tumor volume; TLG, total lesion glycolysis.

Continuous parameters were expressed as mean ± standard deviation with range for normal distribution and included median when the values were non-normal distribution.
